# Refining Program Theory for a Place-Based Integrated Care Initiative in Sydney, Australia

**DOI:** 10.5334/ijic.5422

**Published:** 2020-09-28

**Authors:** John Eastwood, Salwa Barmaky, Sally Hansen, Erin Miller, Suzanne Ratcliff, Penelope Fotheringham, Heidi Coupland, Denise De Souza

**Affiliations:** 1 School of Women’s and Children’s Health, The University of New South Wales, Sydney, NSW, AU; 2 Ingham Institute of Applied Medical Research, Liverpool, NSW, AU; 3 Charles Perkins Centre, Menzies Centre for Health Policy, Discipline of Child and Adolescent Health, and School of Public Health, University of Sydney, Sydney, NSW, AU; 4 Sydney Institute for Women, Children and their Families, Camperdown, NSW, AU; 5 Community Health Services, Sydney Local Health District, Camperdown, NSW, AU; 6 Drug Health Services, Sydney Local Health District, Camperdown NSW, AU; 7 Centre for Research in Education, Torrens University Australia, Melbourne, VIC, AU

**Keywords:** integrated care, critical realism, place-based initiative, design

## Abstract

**Introduction:**

The Healthy Homes and Neighbourhoods (HHAN) Integrated Care Program seeks to enhance vulnerable family engagement with health and social services through a care coordination model. Besides servicing families in Sydney, HHAN has also established place-based initiatives (PBIs) in areas of disadvantage such as Redfern. The Redfern PBI co-locates HHAN with housing, drug and alcohol services, and financial and legal services. This integration aims to facilitate service access and multi-agency support for vulnerable families in Redfern. Hence, this study aims to evaluate for whom, when and why HHAN’s PBI at Redfern works, or not, and what are its outcomes.

**Methods:**

The project utilises critical realist methodology to undertake a qualitative evaluation of the impact of the PBI on clients, services and the community. Purposive sampling was used to identify 21 participants including HHAN clients, HHAN staff and stakeholders (HHAN partners). In-depth, semi-structured interviews were audio-recorded, transcribed, coded and analysed using a context (C), intervention (I), mechanism (M) outcome (O) (CIMO) approach to abductive analysis.

**Results:**

Five key CIMO configurations of the Redfern PBI emerged – whole-of-family involvement, flexibility, trust, building connections and co-location. Whilst each theory had specific outcomes, overall client outcomes included improved access to services, better outlook, empowerment and engagement with services. Service outcomes included increased collaboration and foundation for integration between HHAN and other services. Negative outcomes included lack of full integration, the risk associated with integration and difficulty evaluating impact.

**Conclusion:**

This study successfully refined the program theory for subsequent use in later implementation of critical realist evaluation studies.

## Background

### Introduction

Savic and colleagues have described integrated care as a “holistic care model” that encompasses individual medical, physical and mental health needs, and includes social issues and environment [1]. Place-based interventions (PBIs) are initiatives that use complex partnership networks to implement multiple-component interventions aimed at changing the social and physical environment within a targeted location. Such an approach to integrated care creates a coordinated approach between multiple healthcare providers such as primary care, social services and hospitals, and supports community services. The PBI approach both connects and aligns the different sectors and organisations [2]. Effective integrated care strategies for primary-secondary interface include factors such as communication and information exchange, interdisciplinary teamwork and shared care guidelines and pathways [3]. Integrated care is thus founded on the interconnectedness between social and health issues of an individual and communities. There are many approaches to the design and implementation of integrated care. A co-location model, through PBIs, is one of them.

PBIs co-locate different services and activities such as legal, health, social and housing within the same physical space. The extant literature supports the PBI approach for several reasons. Firstly, it is local physical and social environments that influence an individual’s wellbeing, health, living and learning [4,5]. Secondly, the feeling of being socially linked to an area enhances wellbeing [6]. Local neighbourhood social support systems have been found to be important in maintaining strong social bonds within a community and combating the risk of isolation, which in turn may impact on family stress and functioning [7]. Baum and Gleeson [8] have argued that geographical areas associated with a higher prevalence of social disadvantages – characterised by low school attainment, high incarceration rates and child abuse – may become entrenched in stereotype and inherit intergenerational disadvantage [8].

From early 2014, neighbourhood resident committees in the Redfern public housing estate, had been actively engaged in a co-design process with staff from Housing New South Wales (NSW), Sydney Local Health District (SLHD), the City of Sydney, Police, and local non-government agencies providing services on the estate. Meetings with residents were held where concerns were raised regarding the safety of the estate and the lack of services. Agreement was reached to establish a service “hub” in the basement of one of the buildings. The collaborative PBI was later called Red-Link and was incorporated as an integral component of (1) The Inner-West Interagency Child Health and Well-being Plan [9], The “Health Strong Communities” whole-of-government service system reform, and (2) Local integrated care initiatives including the Healthy Homes and Neighbourhoods (HHAN) Integrated Care Initiative [10]. Those interagency planning initiatives commenced in 2013 with strong leadership from district health and community service agencies [11].

The HHAN design called for the trialling of wraparound care in two PBI projects [10]. All elements of the HHAN design were also to be implemented in the Redfern site: including family identification, care-coordination, “wraparound” service delivery, family group conferences, general practice engagement and support, family health improvement, system change activities, and development of assessment tools such as patient-reported measures and the first cycle of critical realist evaluation. The full nature of those local initiatives was not detailed in the design given that the intention was to ensure that the interventions would be locally developed through community and consumer consultation. In the Redfern location regular meetings were held with the Housing Estate neighbourhood advisory boards and a local steering committee was established. Input from the most vulnerable families on the estate was initially difficult but older family members (grandparents) played an important role in supporting and advising program activities.

The design of Healthy Homes and Neighbourhoods also drew on previous local social epidemiology, and related theory building and program design work [12,13,14,15,16,17,18,19,20,21,22,23]. Those earlier empirical studies consistently identified the physical and social characteristics of “place” as important drivers of health and wellbeing. Importantly social isolation and lost expectations emerged as possible triggers for stress, anxiety and depression in the studies of neighbourhood context and maternal stress. Also identified was the absence of specialist service support mechanisms for front-line practitioners and a need to develop integrated care intervention that strengthened care pathways through collocation of services [10,11,24].

### The Intervention

The Redfern PBI was established in early 2015 and enrolled their first family in June of that year. The site was chosen for testing HHAN “procedures for their acceptability, estimating the likely rates of recruitment and retention of subjects, and the calculation of appropriate sample sizes” [25]. In critical realist methodology, this phase was also important for studying the broader context including for example local structures and activities of practitioners; acceptability of the intervention by consumers, service providers, and the broader layered service system. It was also essential that the initial intervention and program theory be examined and refined prior to moving to the Evaluation Phase.

The Redfern PBI has been able to develop a community-based wraparound intervention in partnership with families. The principal characteristic is a strengths-based, whole-of-family approach that looks at the family unit and the dynamics between family members. Thus the practitioners are able to look at how one activity, for example, attendance at health appointments, may be influenced by social determinants of health, including access to clothing and food.

The wraparound model involves having a clinical care coordinator who develops a trusting long-term relationship with the family through providing a persistent, assertive and challenging approach to understand the family’s problems. The clinical care coordinator is tasked with supporting the family to navigate the service system, and coordinating the multi-agency team of supports required. The delivery of wraparound services is intentionally flexible and families are encouraged to set their own goals. The goals identified are measurable and progress towards achieving goals is monitored.

The co-location of workers enables care-coordinators to draw on specialist skills and services to meet the immediate needs of the referred client and family while developing long term plans for the whole family. Thus immediate support is provided and duplication of services delivery is avoided. The sequencing of the level and intensity of support, according to the family’s identified needs, is intended to ensure trust and “buy-in” of families while reducing failed interventions and wasted resources. The HHAN Program Logic, theorised contextual conditions and program theory have previously been published in this Journal as part of the HHAN series of papers [10].

### Philosophical perspective

The research and evaluation described here draw on the critical realist philosophy of science as elaborated in our earlier manuscripts in this collection [26,27]. In keeping with post-positivist tradition, critical realists draw a distinction between the intransitive domain where reality exists independent of our knowledge of it, and the transitive domain that considers our generation of theories to derive incomplete understandings and knowledge about reality. In so doing, it draws from and offers an alternative to, the more established purist paradigms of positivism and interpretivism.

The critical realist understanding of the world (Ontology) proposes that three inter-related domains make up reality. Those domains are (1) the real—where entities are said to possess structures and mechanisms that have generative powers whether these are actualised or not; (2) the actual—where entities under certain conditions actualise the powers and mechanisms they possess to produce events, but these may or may not be empirically observed; and (3) the observed or empirical—where entities actualize their powers and mechanisms under given conditions to produce events that are observed and experienced [28]. Thus, critical realism does not accept empirical observations as the only domain of reality that needs explanation. It seeks to include explanations about how entities are structured, their mechanisms, and the conditions needed to activate those mechanisms [27].

Thus unlike positivist researchers, critical realists do not restrict their study to structures, processes and mechanisms that are visible and empirically observable. Their main claim that is relevant to the implementation of an intervention, or improvement initiative, is that the intervention is influenced by pre-existing and immediate contextual factors that have contributed to shaping and affecting agents’ (i.e. practitioner or service user) behaviours and decision making [28,29].

The Critical Realist methodological approach used here draws on the work of critical realists [30,31,32,33]. The writing of these authors was used to inform the evaluation design, data analysis, and what might count as context and mechanisms. The methodology, therefore, holds that:

1. while realist social theory focuses on explaining society, it is also amenable to being adopted for smaller-scaled human interactions and activities [34], and within program evaluation [33];
2. social structures are real and causally efficacious [31];
3. mechanisms are ‘that which can cause something in the world to happen, and in this respect mechanisms can be of many different kinds’ [32, p 55];
4. social structures pre-exist present day individuals, providing individuals with conditions for action while at the same time constraining and enabling the kinds of actions they can engage in [30,31];
5. social programs introduced into public institutions or contexts as interventions tend to implicate or modify the mechanisms operating in the pre-existent structure [33].

Adopting this theory, De Souza [33] conceptualised *context*, in a social program, as comprising aspects of structure, culture, agency and relations. Insofar as an integrated care setting is interested in modifying or transforming the organisation of its services, it is possible to suggest that social programs—introduced in public institutions as interventions—tend to implicate the structural, cultural, agential and relational mechanisms outlined in Table 1.

**Table 1:** Context, Mechanisms, Outcomes, modified from [33].

Context	Mechanisms related to	Outcomes

Structure – Institutional/Organisational	roles, practices, resources, processes	(T), (I) or (R) of institutional/organisational structure
Culture – Institutional/Organisational	group ideas and propositional formulations about the institution/organisation	(T), (I) or (R) of institutional/organisational culture
Agency	individual beliefs and reasons for actions or non-action	(T), (I) or (R) of individual agency within the institution/organisation
Relations	maintaining, adjusting or redistributing power/duties/responsibilities	(T), (I) or (R) of institutional/organisational relations

*Note*: a. Transformation (T): indicates mechanisms related to different parts of context, and activated by the social program, are producing some anticipated/desired outcomes. b. Invariance (I): indicates that mechanisms related to different parts of context, undergo changes in attributes irrelevant to transformation or reproduction of the context (e.g. doctor-patient relation remains, though different persons may fill the roles)(cf. Sayer, 1992). c. Reproduction (R): indicates relevant mechanisms related to different parts of context have not been adequately activated by the social program, thereby reproducing outcomes the social program aimed to change.

**Figure 1:**
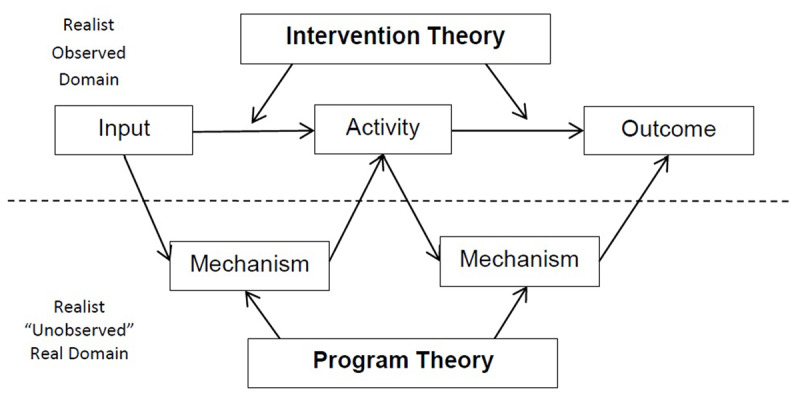
Intervention and Program theory.

In relation to distinction between intervention and programme theory, Blamey and Mackenzie [35] propose that Theory of Change [intervention] theory be used for the purpose of programme planning, improvement and the development of robust monitoring systems at a whole programme level; while realist evaluation be used to examine in detail aspects of the most promising programme (mechanism) theories. Figure 1 illustrates this situating of intervention theory in the “observed” domain and programme theory in the “unobserved” real domain. The eliciting of the explanatory programme theory requires the application of abductive and retroductive logic that seeks “inference to the best explanation” for the observed phenomenon [18].

### Research aims

The study reported here aims to:

1. Elaborate and further define the critical realist case-study
2. Define the historical and current context of the Redfern intervention.
3. Identify, refine and explain the emerging programme theory for the Healthy Homes and Neighbourhoods wraparound, PBI.

## Theory and Methods

### Introduction

**Figure 2:**
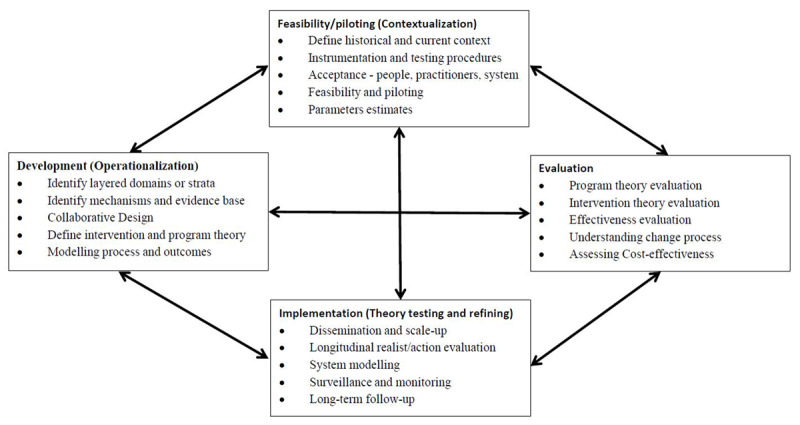
Key elements of the adapted development and evaluation process.

The study reported here is nested within a larger programme of continuous research, design and evaluation. The evaluation approach is informed by the UK Medical Research Council (MRC) Framework for evaluating complex health interventions with its four components, namely 1) development, 2) feasibility/piloting, 3) evaluation and 4) implementation. We have adapted the Framework to include: critical realist and continuous improvement approaches. The underlying critical realist methodology and research framework have previously been described [26,27]. The key elements of the adapted development and evaluation processes are shown in Figure 2.

### Feasibility/piloting (Contextualisation)

The study reported here is part of the feasibility/piloting phase of the MRC Framework and the adapted model above. The MRC Framework describes the purpose of the feasibility and piloting phase as being to test “procedures for their acceptability, estimating the likely rates of recruitment and retention of subjects, and the calculation of appropriate sample sizes”. Missing from the MRC advice is consideration of the need to study the broader context including the acceptability of the intervention by service providers, and the broader layered service system. Within a layered integrated care intervention, the measurement of clinical, process and outcomes indicators is problematic. The causal effects of the social determinants of health on health care outcomes are difficult to evaluate, and are more effective where there is a clear framework that aligns with the community interests and needs. In the adapted framework we have identified the importance of defining the context and instrumentation. The modified Feasibility/Piloting (Contextualisation) elements are:

- Define the historical and current context
- Define instrumentation and testing procedures
- Assess acceptance by people, practitioners, and the system
- Undertake feasibility and piloting
- Determine parameters estimates.

Our critical realist methodology identified the importance of contextualisation of intervention case studies and the subsequent development of data collection tools and approaches in those concrete situations. The integrated care initiative will be evaluated in multiple contexts and system layers. A particular feature of the critical realist approach is the emphasis on studying the historical and current perspective of the layered context. Understanding how over time, the social and institutional organisation of the context has contributed to conditioning and shaping the present-day actions of individuals, are important as they allow the identification and evaluation of the mechanisms involved in driving the individual and community to act in some ways but not others thereby producing certain outcome trajectories. At a clinical level, this involves a comprehensive individual bio-psycho-social interview in a community context. At practitioner, provider and system levels a similar analysis is required. This analysis also has implications for the development of measurement instrumentation in a multi-layered system with the involvement of multiple stakeholders.

### Methodological Approach

A mixed method approach has been undertaken for the overall study and data collection. We used an integrated approach to methods, data collection and analysis. Yin [36] argues that without such integration “different methods may sit in parallel, potentially leading to multiple studies, and not the desired ‘mixing’ of methods implicit in mixed methods research. The methodological approach is described in detail in two earlier papers in this series of papers [26,27]. The study protocol [26] proposed Stage One: Contextualisation of Case Studies; Stage Two: Concretisation and Instrumentation; and Stage Three: Evaluation Data Collection. The focus below is on Stage Three: Evaluation Data Collection.

The purpose of the contextualisation of the case study stage is to focus the evaluation on a limited aspect of the logic model and to study the programme theory mechanisms in detail. In keeping with the critical realist approach taken here, the various contexts will require a description of how the current context came to be so and not otherwise over time. For this purpose, we will use documentary and social epidemiology data sources. The concretization and instrumentation stages of analysis are not reported in this manuscript.

#### Qualitative Data

Qualitative data included: planning documents, meeting minutes, field notes and interviews with clients, clinical staff and other stakeholders involved in the Redfern initiative. The client interviews were conducted either at the RedLink office or within client homes. Stakeholder interviews were conducted at the RedLink office and Croydon Community Health Centre.

#### Participants

Seven clients, 5 clinicians and 10 stakeholders were interviewed. Interviews were carried out between November 2015 to September 2017. The vulnerability of clients at the time of the interview determined whether they would be suitable candidates to interview. Care coordinators of the RedLink Initiative arranged client interviews and through their rapport with these individuals encouraged their participation. Clients were provided with a $50 grocery voucher for their time.

Clinical staff and stakeholders were selected by purposive sampling. Stakeholders were either from services based at Redlink (housing, legal aid, financial support and Aboriginal and Torres Strait Islander services), or other governmental and non-governmental agencies that clients were involved with.

#### Data collection

The interviews were semi-structured and aimed to explore different perspectives of clients, staff and stakeholders associated with the Redfern initiative. The interviews were approximately 30—60 minutes long and were conducted face-to-face by a team of two interviewers. The interviewers had no relationship with the participants prior to the study. The interviewers had a working relationship with the HHAN Team.

Participants were asked about their experience of participating or working with the program, what they thought were its impact on health and social standing (outcomes), and what they believed were the underlying causes (mechanisms) for those impacts. Client-specific questions included those about their backgrounds, reasons for referral (context) and their own personal outcomes, whilst staff and stakeholders were asked specifically about both client and service outcomes. Only one repeat interview was carried out with a HHAN staff member.

The interviews were audio recorded, transcribed verbatim and coded within NVivo version 10.

#### Quantitative Data

At the feasibility/pilot stage we also undertook: a) testing of person-centred reporting measures (PRMs); b) spatial data-linkage studies [37]; c) hospital outcome data-linkage studies [38]; and d) meso-level social networks analysis studies [39,40]. Full reports of those studies will be published separately. Preliminary findings have been previously reported and will be included in this manuscript where relevant and appropriate.

### Analysis and identification of refined theory

#### Analysis

As mentioned elsewhere [18] two predominant approaches to theory building exist in the forms of emergent theory building and confirmatory theory testing. The emergent theory generation phase of theory building moves from empirical observation toward the development of theoretical concepts. The developed theoretical concepts are then organised within the realist social theoretical framework to formulate an explanatory theory. Within critical realism and realist social theory, the framework states that separate accounts—of how a socially structured past comes to bear in the experiential encounters of present-day individuals and how individuals’ actions, in response to these partial encounters, impinge on the structuring of a social future—written from different standpoints are needed [30].

Contextualisation of the case study, to develop an account of the socially structured past, involved drawing on planning documents and minutes of meetings, epidemiological data and information about the service centre embedded within the context of interest. This information was analysed and primarily organised chronologically.

The interview data collected, providing information about the experiential encounters of everyday individuals operating within the social structure, were read and familiarized by one researcher and emerging theories were coded. The coded data were categorized according to context (C), intervention (I), mechanism (M) and outcome (O) as per Table 2. Next, the various theories were grouped into higher and lower order codes. Theories were then organised connecting the outcomes with identified mechanisms that were triggered to generate those outcomes, and finally the contexts within which those mechanisms were triggered. The analysis was informed by both inductive and retroductive modes of reasoning. This was an iterative process undertaken by the second author. Codes were cross-analysed by a second coder in the same team. Areas of contention were discussed at team meetings and once a consensus was reached, the theories were synthesized into this report. The theories were organised as adapted from the layering of reality proposed by Layder [41]. Those layers are: Self – Self-identity and individuals experience; Situated Activity – Face to Face activity; Intermediate Level social and service organisation; and Macro Level social and service organisation [10].

**Table 2:** CIMO-logic – the Components of Design Propositions [42].

Component	Explanation

Context (C)	The surrounding (external and internal environment) factors and the nature of the human actors that influence behavioural change. They include features such as age, experience, competency, organizational politics and power, the nature of the technical system, organizational stability, uncertainty and system interdependencies. Interventions are always embedded in a social system and, as noted by Pawson and Tilley (1997), will be affected by at least four contextual layers: the individual, the interpersonal relationships, institutional setting and the wider infrastructural system.
Interventions (I)	The interventions managers have at their disposal to influence behaviour. For example, leadership style, planning and control systems, training, performance management. It is important to note that it is necessary to examine not just the nature of the intervention but also how it is implemented. Furthermore, interventions carry with them hypotheses, which may or may not be shared. For example, ‘financial incentives will lead to higher worker motivation’.
Mechanisms (M)	The mechanism that in a certain context is triggered by the intervention. For instance, empowerment offers employees the means to contribute to some activity beyond their normal tasks or outside their normal sphere of interest, which then prompts participation and responsibility, offering the potential of long-term benefits to them and/or to their organization.
Outcome (O)	The outcome of the intervention in its various aspects, such as performance improvement, cost reduction or low error rates.

#### Theory Refining

For the analysis undertaken the stratified levels of interest implicated in developing the explanatory theory included self, situated activity, intermediate and macro-levels of social and service organisation. Analytic resolution was used to limit the analysis to the theories that emerged from the theory generation phase. We approached the abductive analysis in three stages. The first step is the abductive inference embedded within the theory generation processes. The second stage, recontexualised the observed phenomena through the lens of theories arising from literature, key informants and the earlier theory generation (such as equitable access to services). Finally, abduction will be undertaken as part of the Inference to best Explanation, as reported here. As the CIMO configurations were developed, we sought confirmatory and contradictory evidence from other sources, including concurrent studies of interagency partnerships, minutes of local meetings, and an external review commissioned by Housing NSW. Divergence of findings was given particular attention as it is here that “new” knowledge or understanding can be elicited through the abductive and retroductive reasoning. Full confirmatory triangulation of findings using other case-studies was not possible in this single feasibility/pilot case-study.

### Ethics

Written informed consent was obtained before the interview with participants reassured of their anonymity. The study was approved by the Research Ethics and Governance Office, Royal Prince Alfred Hospital, Sydney Local Health District (X15-0138 & HREC/15/RPAH/190).

## Results and Discussion

### Contextualisation of the case study

In keeping with the critical realist approach taken here, a description of the historical and current context of the Redfern case-study, from the standpoint of social structure follows.

#### Planning Context 2014

The HHAN Tender proposed a South Sydney Healthy Neighbourhood Support Initiatives using the collaborative use of existing partner-funded services. The tender noted that there was significant locational disadvantage in the South Sydney suburbs of Redfern, Waterloo, Alexandria and Glebe. Much of this was associated with the social and community housing estates in those suburbs. The vulnerable families living in those estates were observed to have perhaps the highest complex health and social needs of any population group in SLHD.

At the time of writing the proposal, there were several initiatives to develop PBI for vulnerable families. Core partner agencies including SLHD (Community Health, Drug Health and Mental Health), FACS (Housing and Community Services), Sydney Day Nursery, Education and Communities (Glebe and Alexandria Park Public Schools) and The Benevolent Society were actively working on new initiatives in those communities. The SLHD Partnership with the Redfern Aboriginal Medical Service had also recently resulted in a stronger collaboration with the development of shared service delivery models.

The tender document argued that there was strong justification for working with families in South Sydney but observed the local context is complex and implementation will have challenges. Opportunities to progress local partnerships had emerged, at that time, through:

1. Partnership discussions with the Redfern Aboriginal Medical Service regarding child and family health services
2. Recent referrals from Jarjum College (Redfern) for Aboriginal children with special needs
3. Outreach paediatric clinics with Our Lady of Mt Carmel Catholic Primary School, and Glebe and Alexandria Park public schools
4. Shared clinics for vulnerable families with The Benevolent Society (Rosebery)
5. A joint City of Sydney and Housing planning initiative for the Redfern Social Housing Estate.

#### Social Epidemiology

Redfern is a suburb within the City of Sydney Local Government Area (LGA) and has historically had high rates of social and economic disadvantage, substance abuse, domestic violence and mental illness within its community. The demographics of Redfern are rapidly changing, with increasing affluence and decreasing rates of crime and drug use. Redfern is also a political and social centre for the urban Indigenous population of New South Wales. In the 2011 Census, there were 13,213 people in Redfern, of which 2.4% were of Aboriginal or Torres Strait Islander background, compared to 1.2% in Sydney and 2.5% nationally [43] The Census data, however, are believed to underestimate the extent of Redfern’s Aboriginal community, since many people may refuse to fill out forms or declare Indigenous status, and the proportion of Redfern-Waterloo’s Indigenous population has been estimated by members of the Aboriginal community to be twice that of the national figure of 2.5% [10,12,13].

**Figure 3:**
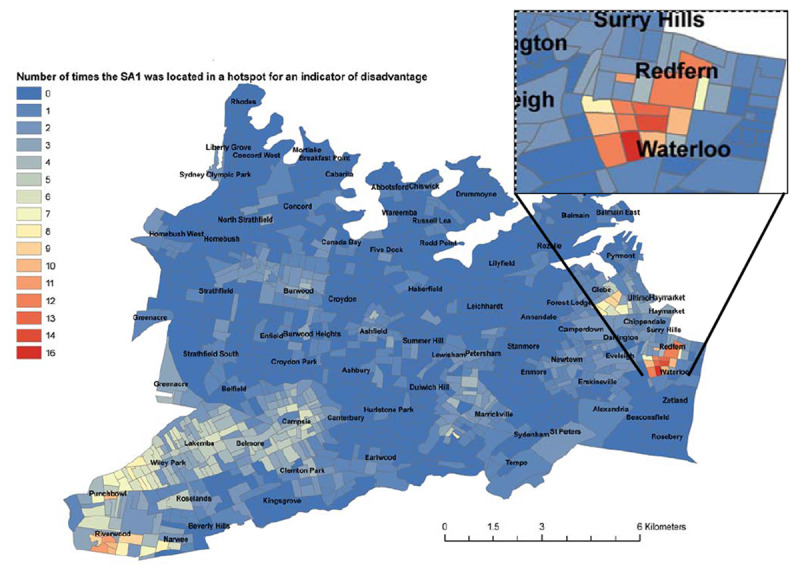
Geospatial analysis of perinatal family stress – SLHD, 2014.

As a component of the Feasibility/Pilot Phase of the Healthy Homes and Neighbourhoods Evaluation, we undertook Geospatial analysis of perinatal family stress and social disadvantage reflecting family disadvantage in the SLHD. The full analysis will be reported separately. The purpose of the analysis was to assist implementation planning and to provide baseline analysis for the longitudinal evaluation. That analysis was used to identify those communities that might benefit from a PBI. Markers of vulnerability and disadvantage that were related to areas such as unemployment, housing and perinatal risk factors were collated to create an algorithm for family disadvantage. Redfern and Waterloo were found to have the highest family disadvantage level as shown in Figure 3.

#### RedLink Integrated Service Centre

SLHD and NSW Family and Community Services – Housing partnered in 2015 to establish an Integrated Service Centre that was subsequently called RedLink. The HHAN Integrated Care initiative placed two clinical staff in RedLink and it was from this base that they provided the wraparound intervention for enrolled clients.

A Service Level Agreement (SLA) was initiated prior to the commencement of RedLink in mid-2015, which articulated the vision, objectives, governance structure and expectations for service partners during the first 12 months of operation. The SLA stated that RedLink will: a) Provide community, health, housing, legal and other support services to residents of the public housing estate in Redfern from its base in the McKell building, and b) Jointly develop a range of community activities and engagement strategies to improve community wellbeing and build long-term relationships within the community.

Key service partners include: a) FACS Housing; b) SLHD (including: Healthy Homes and Neighbourhoods, Drug Health Harm Minimisation, Community Mental Health, Chronic and Complex Care Integrated Care, Aboriginal Chronic and Complex Care); c) Legal & Financial Services (including: Legal Aid NSW, Redfern Legal Centre, Good Shepherd Financial Counselling); and Local Government, non-Government Organisation and local community organisations.

### Theory Refining

#### Theory 1 (Level of situated activity): Whole-of-family involvement intervention approach (Table 3)

**Table 3:** CIMO propositions for Whole of Family Involvement intervention.

Present contextual mechanisms activated produce the following outcomes [C_M_]	Proposed Intervention Design Elements [I]	Postulated Intervention Programme Mechanisms activated at the Level of Situated Activity [M_P_]	Postulated psychological, motivational and behavioural Outcomes [O]

– Intergenerational trauma – Children being assumed into care – Disconnection from services – Significant Aboriginal and Torres Strait Islander people as clients of HHAN	– Whole of family involvement – Families participating in decision making – Fostering trust and collaboration between services, HHAN and families Cultural sensitivity	– Trust	Client outcomes: – Increased family stability – Increased family cohesion – Establishment of greater trust in services

The planned intervention in the programme design was “friendship and family support”. It became apparent from the interviews that the “whole of family” approach taken by the clinicians was unique when compared to other services operating, in the past and currently, within the RedLink service site. The intervention was characterised as a focus on adult family members in addition to the children. In addition, the focus on adult family members often extended to include grandparents and other relatives. The approach of ‘Whole-of-family involvement’ is an important program element recognised by interviewees in the context of many Redfern residences being impacted by intergenerational trauma, especially for Aboriginal and Torres Strait Islander people. Also, clients and stakeholders described harrowing experiences of families with children in out of home care. Moreover, many clients have previously been disconnected with services, particularly after being disenfranchised from a system that had offered little help until that point.

Oftentimes, the available pre-existent services were limited in dealing with families as a unit. One stakeholder expressed that their service did not understand the issue of child welfare and other child-related concerns because “we’re the service that focuses on the adult … our facility is not set up to see children”.

A clinician had this to say about whole-of-family care:

Interviewer: And then just moving onto the Healthy Homes team…What gap do they plug?

Clinician: Totally the family…isolated, lone people who were quite disenfranchised, disconnected and broken in many ways…

In this CIMO theory, targeting the level of situated activity, the HHAN intervention design element of “whole of family approach” enabled the activation of mechanisms that involved families in decision-making. The conditions needed to actualise co-decision-making practices included developing trust (mechanism) and collaboration (mechanism) between services, HHAN and families to realise positive outcomes. Stakeholders suggested that cultural sensitivity was another necessary condition to establish to reach out to culturally diverse clients such as Aboriginal and Torres Strait Islander people. Five out of ten stakeholders found ‘whole-of-family involvement’ invaluable.

Resultantly, stakeholders report outcomes of increased household stability and happier families:

Healthy Homes…have created happy households…Healthy Homes wraps around the whole family and then supports and re-supports … Healthy Homes and Neighbourhoods have got access to families and their total family network that no other part of health has and no other part of FACS [Family & Community Services] has.

Stakeholders have also found that through HHAN clients’ ‘trust and rapport with health has increased’.

#### Theory 2 (Level of situated activity): Flexibility of delivery intervention approach (Table 4)

**Table 4:** CIMO propositions for flexibility of service intervention.

Present contextual mechanisms activated produce the following outcomes [C_M_]	Proposed Intervention Design Elements [I]	Postulated Intervention Programme Mechanisms activated at the Level of Situated Activity [M_P_]	Postulated psychological, motivational and behavioural Outcomes [O]

Client characteristics: – Complex health and social needs – Distrust of previous services – Disconnection from services – HHAN programme’s characteristics	Flexible programme model: – No strict referral criteria – No prescriptive evaluation tool – No time frame – Prioritise client need rather than service objectives – Clinician autonomy and adaptability	– Flexibility	Client outcomes: – Better engagement in services – Improved outlook andplanning for the future

Flexibility emerged as a key mechanism underlying the context of client vulnerability. Issues faced by some of the clients interviewed include poverty, unemployment, drug and alcohol addiction, gambling, moderate-to-severe mental health conditions, single parenthood, traumatic childhood experiences, experiences of violence, experiences of grief and loss, and homelessness. Resultantly, clients reported struggles with accessing services. A client with experiences of homelessness and severe mental illness described this issue:

Interviewer: Would you still have been able to engage with them so well if they [HHAN staff] hadn’t come to you?

Participant: No, I couldn’t…I just shut my phone and locked myself in there.

Stakeholders also reported that clients felt disconnected from services, and many had fallen through the cracks.

HHAN’s flexible programme model also formed the context for this theory. HHAN’s programme has no strict referral criteria, prescriptive evaluation tool nor time frame for discharge.

Stakeholders identified ***flexibility*** as the mechanism (M) that caused the outcomes. The programme modalities were prioritising client need rather than service objective, and clinicians having autonomy being adaptable. Here is a quote from one of the clinicians that convey this theory best:

Every service has their scope and we’re basically saying…we’ll take on these families with children up to 17 and identify all these broad issues and get as many services as we can for the best interests of the child. And…that’s unique, I haven’t seen that anywhere.

Five out of ten stakeholders have reported that flexibility is a key feature for HHAN’s success. Therefore, the outcome from flexibility was that clients engaged with services more and improved their outlook on life and planning for the future. A client stated after HHAN had helped her, she wanted to pursue further goals such as enrolling in a TAFE course, getting her drivers’ license and moving to a new house.

#### Theory 3 (Level of Situated Activity): Focus of the intervention on building trust (Table 5)

**Table 5:** CIMO propositions for Intervention focus on building trust.

Present contextual mechanisms activated produce the following outcomes [C_M_]	Proposed Intervention Design Elements [I]	Postulated Intervention Programme Mechanisms activated at the Level of Situated Activity [M_P_]	Postulated psychological, motivational and behavioural Outcomes [O]

– Distrust and disconnection from previous services – The power imbalance between client and service provider	– Building rapport and responding to need – Attentive to client concerns – Flattening the power base – Informal client-clinician interactions – Non-judgemental interactions – Favourable interpersonal skills	Trust	– Empowerment for clients – Establishment of greater trust in services for clients – Better engagement in services for clients

The context for this CIMO theory is very similar to programme flexibility. Participants report distrust and disconnection from services. Stakeholders reported that this distrust in the Redfern community was perpetuated by a perceived fear of being judged by services, power imbalance inherent in the service provider-client relationship, and apathy with regards to how much they could actually help. This is a quote from a client encapsulating how many of their clients feel:

Even if I’ve been made a doctors’ referral and I have to go like I’m always a little bit nervous walking into a room with a stranger and telling them personal information about myself. To expect someone that has vulnerabilities to go and find their way…it doesn’t work.

Several conditions were established to activate feelings of trust that helped to produce positive outcomes for clients. Firstly, HHAN clinicians established trust by building rapport and responding to client need:

Interviewer: How do you feel about them? Do you trust them?

Participant: It’s good and the point is you don’t have to worry too much about how you’re seeming to them, you can just be yourself, and they will try to help in the best way they can.

Five out of 8 clients felt that HHAN had built a good rapport with them.

Secondly, HHAN clinicians also tried to establish interactional conditions that flattened the power base, engaging in informal conversations, meeting in a space where participants were more likely to feel comfortable rather than a formal clinical setting, and paying full attention to them through every small interaction. In one example a clinician mentioned was ‘they remember the children’s names and so there’s that interaction… and that makes them feel good because someone likes my child, someone’s giving them attention’. Furthermore, participants noted that the establishment of trust was dependent on highly experienced clinicians with exceptional interpersonal skills.

The close attention to clients circumstances activated feelings of trust (mechanism) that resulted in outcomes such as engaging clients that were difficult to engage with prior to HHAN, improving their outlook on life and greater trust in services. A client positively recalled that as a result of HHAN ‘I can see good … I can go places, I can be somebody’.

#### Theory 4 (Intermediate level of social and service organisation): Intervention focus on building connections (Table 6)

**Table 6:** CIMO propositions for connecting services intervention.

Present contextual mechanisms activated producing the following outcomes [C_M_]	Proposed Intervention Design Elements [I]	Postulated Intervention Programme Mechanisms activated at the Intermediate Level of Social and Service organisation [M_P_]	Postulated psychological, motivational and behavioural Outcomes [O]

– Silo-working – Complex health system – Clients having complex health and social needs – Services having little understanding of the health system – Competing priorities – Services’ distrust of other services	– Connecting services – Informal and formal communication – Employment of experienced clinicians – Interpersonal skills of clinicians – Recognition of established networks rather than creating new systems	– The motivation of clinicians and services to collaborate	Positive outcomes: Clients: – Better engagement in services for clients Services: – Increased collaboration between services – Foundations for the integration of services – Improved relationship between HHAN and other services – Avoid service duplication – Introduction of new health services to Redfern
		– Confidentiality and privacy concerns over sharing data about clients and integrating systems – The unwillingness of organisations to take a risk or change current structures to integrate	Negative Outcomes Services: – Systems not yet integrated – Difficulties with information sharing

The theory of ‘building connections’ aimed to address the context of the organizational culture of *services*, rather than clients. Participants opined that services were siloed, operated in isolated spheres and did not communicate. As clients’ situations are complex, this structure made it easy to overlook details and be unaware of other services involved. Furthermore, services had competing priorities, which impeded collaboration. Stakeholders and HHAN clinicians also reported services were also less aware of workings of other sectors, such as social services finding it difficult to help clients navigate the complicated health system. For example:

If the system worked properly and people communicated and trusted each other and nobody had these big professional guards up… we wouldn’t need a care coordinator to come in.

In this context, stakeholders and staff reported the importance of the mechanism of formal and informal communication to facilitate better integration of services at Redfern. This theory encompassed getting services to communicate with one another about shared clients, through organising meetings, follow-ups on clients’ various appointments with different services and ensuring that everyone was up-to-date on their progress. This was found to be aided by the clinicians’ good interpersonal skills, participants noted.

Conjointly, there was also an understanding that many networks already existed, and instead of providing a new service, the current system was bolstered through this collaboration. This resulted in an attraction of more health services to Redfern, such as drug health, chronic disease and mental health.

This theory was only possible through mechanisms of motivation for both HHAN and services to collaborate together.

A housing services officer had this to say about their relationship with HHAN:

I didn’t realise how Health works [sic] before. You know I do and I think it’s vice versa…It’s understanding the workings of each other’s organisations have been a big, big help.

Ultimately, the outcome of these connections is a step closer to the integration of services at Redlink, in government sectors and NGOs working with the same client. Additionally, 6 out of 10 stakeholders have acknowledged an improved understanding of other services and their own clients and found it easier to refer these clients to these new services. Resultantly, clients have also been better engaged.

It is in this same context, however, that negative outcomes have also developed due to two particular mechanisms. These mechanisms include, firstly, providers expressing reluctance to share data due to confidentiality and privacy concerns. Participants also reported unwillingness for some organisations to take the risk or change their structures to integrate.

*A lot of people have this sense of hero mentality. I want to be the hero or save a family, they don’t trust someone else to work on their client, so they don’t work together. So when we say, ‘hey let’s co-ordinate things’, everyone’s like ‘whoa, we do it fine, we don’t need you’ (Clinician Practitioner)*.

Thus, the outcome of these mechanisms at the service level has been difficulties with information sharing and lack of total system integration.

#### Theory 5 (Macro-level of social and service organisation): Co-location (Table 7)

**Table 7:** CIMO propositions for co-location of services intervention.

Present contextual mechanisms activated [C_M_]	Proposed Intervention Design Elements [I]	Postulated Intervention Programme Mechanisms activated at the Macro-Level of Social and Service organisation [M_P_]	Postulated psychological, motivational and behavioural Outcomes [O]

– Redfern as a hotspot for vulnerability – Disconnection from community	– Co-location – Accessibility for clients – Proximity – Recognition by clients of Redlink hub as part of the community	“crossing of paths” Increased personal interaction	Positive outcomes Clients: – Better access to services – Better engagement in services for clients
– Complex health, social, economic and environmental issues – Siloed and complex health system – Complex health, social, economic and environmental issues – Siloed and complex health system	– Co-location – Engagement of a diverse range of services	– communication between services – collaboration (e.g. acceptance of shared risk) – Flexibility (e.g. accommodate diverse objectives)	Positive outcomes: Services: – Better integration of services – Improved relationship between HHAN and other services Negative outcomes Services: – Difficulty evaluating the impact of HHAN/difficulty determining discrete impact/outcomes of HHAN – Reception of HHAN dependent on the reputation of all services at Redlink

Co-location operates in the contextual background of Redfern being an area of heightened disadvantage, due to the location of social housing and many vulnerable individuals with complex needs. This has resulted in issues such as tensions amongst cultural groups, environmental health issues with poor housing conditions, and, more recently, strain from the rising affluence from a portion of the neighbourhood:

Interviewer: Why do you think people aren’t linked to services?

Clinician: I think my personal opinion is that the area is becoming quite identified. Now you know the café is up along the way or fancy cafes and there’s, there are just a different people living here. A lot of feel like the estate is closing in and they are not wanting to expand out.

Hence, due to the rising inequality, clients feel unwanted and disconnected from the community. The siloed and complex health system has formed an additional barrier, and both these contexts have resulted in poorer help-seeking behaviour, as reported by clients and stakeholders.

Thus, the causal mechanism activated within these above-mentioned contexts is perceived accessibility for clients, as a result of its proximity to Redfern social housing estate. Furthermore, Redlink has been accepted by clients as part of the community.

Stakeholders have remarked that the program modality of ‘co-location’ of services has resulted in positive outcomes: Redlink as a platform for communication and collaboration between services, engagement of a diverse range of services (housing, legal, financial, health), acceptance of shared risk by services at Redlink and flexibility to accommodate diverse objectives for their clients. A stakeholder has the following to say about this collaboration:

The collocation is extremely beneficial. I should explain the quality of work you see, and the collocation is a big one in partnership and integration. It means that we are all talking Housing, Partners in Recovery, Drug Health and mental health, we are **all crossing paths** very often and we weren’t doing that before.

The proximity and frequent **crossing of paths** (mechanism) resulted in positive outcomes for both services *and* clients. Clients acknowledge that the proximity to Redlink has improved access to services, including HHAN, many of whom professed they would not present for appointments had they lived far away. Services are now a step closer to integration and HHAN’s relationship with them has improved.

However, the co-location of services results in difficulty evaluating outcomes as solely being due to HHAN without evaluating Redlink entirely. On this, a HHAN staff member said, ‘I think in Redfern yes, there is huge community outcomes there, but I don’t think that’s because of HHAN, I think it’s Redlink.’

Also, HHAN staff are concerned that integrating HHAN with services at Redlink may have clients conflating the actually different services as one. The reception and engagement with HHAN will then be dependent on the reputation of Redlink.

## Discussion

This study has provided evidence for the efficacy of HHAN’s PBI in Redfern. The 5 main theories operating in HHAN’s Redfern PBI are whole-of-family involvement, programme flexibility, establishing trust, building connections and increased interaction (“crossing of paths”).

Under the contexts that vulnerable families experienced intergenerational trauma, disconnection from services and having children assumed into care, a whole-of-family approach – requiring families to participate in decision making – tended to produce positive outcomes of increased family stability. Studies show that intergenerational trauma is common in vulnerable populations, due to the inheritance of disadvantage from parent to child and the interconnected nature of the nuclear family [44]. However, family-centred care has the potential to reduce these cycles of intergenerational trauma [45]. To target these cycles, HHAN involved families in decision-making and focused on cultural sensitivity. Therefore, when designing programmes for vulnerable populations, we need to look beyond the individual and see the family as the basic unit of a community. Programmes should also understand their population demographics and cultural factors to deliver culturally-appropriate care.

Within contexts of vulnerable peoples’ complex needs and disconnection from services, mechanisms of flexibility – requiring clinicians to prioritise client objects and be adaptable – produced positive outcomes of improved outlook and engagement with services. Old literature characterises vulnerable populations’ as ‘hard-to-reach’ owing to their refusal to engage with services [46]. Traditional causes include transport difficulties, lack of time, lack of awareness of existing services, language barriers, mental and physical health impedance, whilst larger, sociocultural factors include fear of judgment due to stigma, differences in race, culture and age [47]. Contrastingly, HHAN’s flexible programme focuses on how its service can remove barriers to improve *access*, which is supported by the literature as being an effective component of initiatives that work with ‘hard-to-reach’ populations [48].

For HHAN, the theory of flexibility is expressed in numerous instances: firstly, the programme has broad referral criteria, encompassing families with a vulnerability (a term loosely defined on a case-by-case basis), with children under the age of 18. This means that fewer families are turned away. Furthermore, HHAN also has no prescriptive evaluation tool. The literature says that these evaluations have been found to alienate those with poor experiences with services [49].

Moreover, clinicians work autonomously *for* the client, unlike prior services that follow strict criteria and protocol. However, clinician flexibility is also dependent on the experience and problem-solving ability of the clinician. Therefore, when working with vulnerable populations, a programme needs to be flexible enough to engage with the clients with vulnerabilities, complex needs and tumultuous situation.

In the context of distrust, disconnection and power imbalances between client and services, mechanisms that built trust – such as responding to need and flattening power base – had positive outcomes of increased client empowerment, trust and engagement with services. The literature finds that vulnerable populations have difficulty trusting people and institutions due to negative experiences, and correspondingly present less to services [50,51,52,53]. Additionally, power imbalances between vulnerable populations and services exist, especially between Aboriginal and Torres Strait Islander populations and Western healthcare organisations [54]. However, establishing trust in providers is imperative in improving access, as people with high levels of trust are more likely to seek care when in need, present more often and will adhere to and return for follow-up [55,56,57]. Thus, mechanisms of responding to client need and being attentive to their concerns were found to build trust. The programme also tried to flatten the power divide via informal conversations, meeting in a non-clinical space and active listening. These actions were also found in other programmes to also develop trust [58]. In essence, programmes need to recognise that vulnerable populations find it challenging to trust, and feel unequal power distributions keenly, which are often imperceptible to providers. To counter this imbalance and distrust, our research suggests the key factors that help are responding to need and lessening the divide through subtle interactions.

Two CIMO configurations emerge in Theory 4. Firstly, under contexts of a siloed, complex health system, mechanisms that built connections between services – including communication and motivation to collaborate – produced positive outcomes such as improved collaboration and a foundation of integration between HHAN and other services, ultimately improving engagement with clients. The silo-working of services is well known, especially in health systems [59,60]. Due to the complexity of the health system, resources, boundaries, privacy legislation can cause problems with collaboration. To combat this, information sharing via a service integration model has been shown to improve accessibility to services for clients [59,61,62]. Hence, HHAN drew services to communicate with each other to reduce this silo-working culture, by encouraging formal and informal interactions. This was facilitated through mechanisms of employment of experienced clinicians with excellent interpersonal skills.

But within these same contexts, as a result of mechanisms such as an unwillingness of organisations to take the risk or change current structures, systems are not yet fully integrated. The literature demonstrates that similar to how vulnerable people struggle to trust services, services have trouble trusting each other[63]. Also, differences in skills, culture and working conditions between professions can impede joint working [64]. Besides these issues, for HHAN, services raised confidentiality and privacy concerns over data sharing about clients. Resultantly, integration of service systems at Redfern is not complete. In summary, future programmes should note that to tackle complex, siloed systems, building connections through communication, information sharing and employment of skilled clinicians produce positive outcomes and lay the groundwork for integration. However, full-service integration requires sustained effort, as cultural shifts within organisations to realise the benefits of integration take time.

Lastly, within contexts of Redfern having areas of vulnerability, mechanisms of co-location found positive outcomes such as improved access for clients and increased collaboration between services. Studies find that co-location of a range of services allows for increased relevance and accessibility for vulnerable and disadvantaged families [65]. Furthermore, better programme outcomes occur when there exists meaningful and regular contact with service providers and incorporating local conditions into the programme design [64].

The mechanisms of Redlink as a platform for communication between services, however, may result in negative outcomes. It is tough to evaluate the impact of HHAN as it is challenging to differentiate outcomes of HHAN discretely from that of services at Redlink. This is because designing an effective evaluation tool for integrated mechanisms that purely assesses one component is very difficult [25]. Additionally, the reception of HHAN may be dependent on the reputation of all services at Redlink, as clients may not differentiate between services in an integrated programme. Hence, negative perceptions of other services may affect engagement with HHAN. Thus, future programmes should understand that risks of co-location include difficult evaluation of outcomes and shared risk.

## Strengths and weaknesses

The team of coders agreed that saturation of emerging theories from the 21 interviews was accomplished, achieving theoretical completeness and adding strength to the conclusions reached [66]. We also found that realist methodology was found to be applicable for programme evaluations such as this one. Theorising CIMO configurations allowed a deep understanding between the different aspects of HHAN, a complex intervention with multiple variables [67]. Lastly, a broad range of data from multiple perspectives was used.

However there are also limitations to the findings that we report. The coding and CIMO analysis are abductive and retroductive analytical modes of reasoning. Eco’s typology of abduction includes over-coded, under-coded and creative types of abduction (Eco, 1984 as cited by [32, p 23]. Over-coded abduction is a mode of inference consisting of spontaneous interpretations based on cultural and social prejudging. Thus, all observations involve some form of interpretive abductive process being a precondition for the observed phenomenon having any meaning at all. This interpretive abduction occurs naturally during realist interviews, initial coding of qualitative data, and possibly also in the CIMO analysis.

Secondly, clients that participated in the interviews likely already had a positive bias for HHAN. Similarly, researchers’ relationship with HHAN staff might also cause bias in the data. Furthermore, interviews might not reflect the programme in its entirety as some were conducted when HHAN was being properly established. As interviews were conducted as part of an overall evaluation of HHAN, they might not all be reflective specifically of the Redfern PBI part. Also, this study was unable to comment on the healthcare outcomes of HHAN.

## Conclusions

This study has important implications for developing PBIs for vulnerable populations, and more broadly for integrated care initiatives, filling the existing gap in the literature. The application of critical realist methodology to evaluate this intervention has been effective in identifying the context, mechanisms and outcomes in a complex PBI, despite the limitations of the study cited above. The findings have further defined the historical context of distrust, disconnection and power imbalances that have existed between the client and services in the Redfern area, and allowed us to identify the context and mechanisms of the HHAN program in its early stages that counter this historical context.

It has refined and further explained HHAN’s programme theory, that family-focussed, PBIs are useful in meeting the needs of vulnerable populations and improving collaboration between siloed services. Whilst many of the theories of the PBI that emerged – whole-of-family care, flexibility, trust, building connections, interaction – may seem implicit and obviously useful for vulnerable populations, many integrated programmes are still in their infancy, especially in Australia. Therefore, this study aims to be part of a cultural shift changing the way services can work with and ultimately impact their clients.
